# The glycoprotein-hormones activin A and inhibin A interfere with dendritic cell maturation

**DOI:** 10.1186/1477-7827-6-17

**Published:** 2008-05-06

**Authors:** Sabine E Segerer, Nora Müller, Jens van den Brandt, Michaela Kapp, Johannes Dietl, Holger M Reichardt, Lorenz Rieger, Ulrike Kämmerer

**Affiliations:** 1 University of Würzburg, Department of Obstetrics and Gynecology, Josef-Schneider-Straße 4, 97080 Würzburg, Germany; 2 University of Würzburg, Institute for Virology and Immunobiology, Versbacherstraße 7, 97078 Würzburg, Germany; 3 University of Göttingen, Medical School, Department of Cellular and Molecular Immunology, Humboldtallee 34, 37073 Göttingen, Germany

## Abstract

**Background:**

Pregnancy represents an exclusive situation in which the immune and the endocrine system cooperate to prevent rejection of the embryo by the maternal immune system. While immature dendritic cells (iDC) in the early pregnancy decidua presumably contribute to the establishment of peripheral tolerance, glycoprotein-hormones of the transforming growth factor beta (TGF-beta) family including activin A (ActA) and inhibin A (InA) are candidates that could direct the differentiation of DCs into a tolerance-inducing phenotype.

**Methods:**

To test this hypothesis we generated iDCs from peripheral-blood-monocytes and exposed them to TGF-beta1, ActA, as well as InA and Dexamethasone (Dex) as controls.

**Results:**

Both glycoprotein-hormones prevented up-regulation of HLA-DR during cytokine-induced DC maturation similar to Dex but did not influence the expression of CD 40, CD 83 and CD 86. Visualization of the F-actin cytoskeleton confirmed that the DCs retained a partially immature phenotype under these conditions. The T-cell stimulatory capacity of DCs was reduced after ActA and InA exposure while the secretion of cytokines and chemokines was unaffected.

**Conclusion:**

These findings suggest that ActA and InA interfere with selected aspects of DC maturation and may thereby help preventing activation of allogenic T-cells by the embryo. Thus, we have identified two novel members of the TGF-beta superfamily that could promote the generation of tolerance-inducing DCs.

## Background

Successful pregnancy depends on an adequate endocrine response [1]. Apart from several steroid hormones such as progesterone, estradiol and cortisol, also a number of glycoprotein-hormones are secreted by the fetoplacental unit in early human pregnancy [2]. Amongst those, two members of the transforming growth factor beta family (TGF-β), namely activin A (ActA) and inhibin A (InA), are present in the serum at dynamic levels during the first trimester of human pregnancy [3]. Both hormones are locally produced by the human placenta and fetal membranes, which results in serum levels in the low ng/ml range peaking at 10 weeks of gestation [4] ActA and InA serve as useful diagnostic markers. ActA serum levels are detectable in the sera of pregnant women starting at the time when a pregnancy test is found to be positive. Therefore, it has been speculated that ActA is an important mediator of early embryonic as well as placental development and decidualization [5]. Consequently, decreasing ActA levels correlate both with an ongoing miscarriage and ectopic pregnancy [6]. Increased InA levels allow prediction of pregnancy following in vitro fertilization [7] while decreasing InA levels are used as a specific marker to reveal preclinical abortions [8,9].

In parallel with rising hormone levels, a significant increase in leukocyte numbers is observed in the decidua of early pregnancy. Amongst those, CD14^+ ^monocytes are a predominant cell population [10]. Following activation by cytokines and growth factors, CD14^+ ^monocytes can differentiate into CD209^+ ^immature dendritic cells (iDCs), which are especially efficient in antigen uptake and presentation. Exposure to maturation stimuli that originate from infection or inflammation induces transformation of iDCs into mature DCs (mDCs) that express costimulatory molecules such as CD40, CD80, CD83 and CD86 [11]. There is evidence that iDCs may contribute to the establishment of peripheral tolerance in the absence of such signals [12]. Additionally, it has been shown that iDCs have the capacity to modulate regulatory T cells [13]. Both, in the non-pregnant human endometrium and the decidua of early pregnancy, only a few CD83^+ ^mDCs are detected. In contrast, the number of iDCs is strongly increased at this stage [14]. Therefore it has been suggested that iDCs are important mediators of the immunological tolerance, which is indispensable for successful pregnancy [15].

Up to now, several factors have been proposed which could induce iDCs to adopt a tolerance-inducing phenotype and thereby prevent further maturation of these cells. Amongst those are glucocorticoids, which are highly potent modulators of the immune system. Indeed, several studies have revealed that glucocorticoids are able to impact the function of monocyte-derived DCs and to bias them towards a tolerance-inducing phenotype [16,17]. Following exposure to Dexamethasone (Dex), a synthetic glucocorticoid, DCs acquire the ability to convert the local cytokine pattern to support T_H_2 responses [18,19]. Dex-treated DCs are resistant to maturation induced by lipopolysaccharide (LPS) or CD40 ligand and produce high amounts of interleukin 10 (IL-10) [20]. TGF-β1, another factor known to exert immunosuppressive effects, was found to inhibit the maturation of DCs and to concomitantly induce the generation of tolerogenic DCs [21,22]. Moreover, exposure of TGF-β1-pretreated cells to maturation stimuli prevented up-regulation of CD86 and CD83, classical features of mature DCs [23]. Since ActA and InA are members of the TGF-β family, we speculated that these glycoprotein-hormones might also exert immunosuppressive functions and thereby inhibit the maturation of iDCs in the human pregnancy decidua. Thus, we examined the influence of ActA and InA on the maturation and function of DCs. Our studies revealed that both hormones exert potent effects on at least some features of DCs that partially resemble those seen after Dex or TGF-β1 treatment. Thus, glycoprotein-hormones such as ActA and InA are likely candidates to contribute to the peripheral tolerance seen in the human pregnancy decidua.

## Methods

### Dendritic cell preparation

Buffy coats were obtained from healthy male and female human donors (University of Würzburg, Department of Immunohematology and Transfusion Medicine, Germany). Cells were isolated and cultured as described by Romani et al. [24]. After dilution with an equal volume of phosphate buffered saline (PBS), citrate buffered buffy coats were distributed over a density-gradient (density 1.077 g/ml, leukocyte separation medium, PAA, Cölbe, Germany) and centrifuged at 400 g for 30 min at room temperature. Peripheral blood mononuclear cells (PBMC) were collected from the interface and washed with PBS, resuspended in AIMV-Medium (Gibco, Invitrogen, Karlsruhe, Germany) and incubated for 1 h at 37°C with gentamycine (20 μg/ml; Biochrom, Berlin, Germany) and 2% autologous plasma on plastic dishes (TPP, Trasadigen, Switzerland). To remove non-adherent cells, the dishes were thoroughly washed several times with PBS. The remaining adherent cell fraction was used for the generation of monocyte-derived DCs by culturing them with 800 U/ml purified recombinant human granulocyte macrophage colony-stimulating factor (GM-CSF; Leukomax, Brussels, Belgium) and 250 U/ml IL-4 (Immuno-Tools, Friesoythe, Germany) for 5 days in RPMI 1640 (Biochrom) with 10% fetal calf serum (FCS: PAN, Aidenbach, Germany) and 20 μg/ml gentamycine. On day 3 half of the volume of medium was replaced by fresh medium and IL-4/GM-CSF.

Maturation of DCs was induced on day 4 by adding an inflammatory cocktail containing 1000 U/ml purified recombinant human IL-1β, 1000 U/ml purified recombinant human IL-6, 1000 U/ml purified recombinant human tumor necrosis factor alpha (TNFα) (all Immunotools, Friesoythe, Germany) and 10^-8 ^mol/l prostaglandin E_2 _(PGE_2; _Calbiochem, Bad Soden, Germany) for 24 hours resulting in mDCs. To obtain iDCs for control, an aliquot of the cells remained in culture for another 24 hours in IL-4/GM-CSF medium without addition of the maturation cocktail. All cells were harvested on day 5 for further experiments.

### Hormonal treatment of DC cultures

ActA, InA, TGF-β1 or Dex were added at the following concentrations to the cultured cells on day 4 in parallel with the maturation-inducing cytokines: 100 ng/ml ActA, 100 ng/ml InA, 4 ng/ml TGF-β1 and 10^-6 ^M Dex. All hormones were used in water-soluble form. TGF-β1 was obtained from Biozol (Eching, Germany), ActA from tebu-bio (Offenbach, Germany), InA from diagnostic systems laboratories (Webster, UK) and Dex from Sigma-Aldrich (Deisenhofen, Germany).

For analysis, the cells were harvested after 24 h of culture.

### FACS Analysis

Flow cytometry of DCs was performed using FITC-labeled specific antibodies and the respective isotype controls: CD40 (clone: 5C3, Pharmigen, Heidelberg, Germany), CD83 (clone: HB 15a; Beckman/Coulter, Krefeld, Germany) and CD86 (clone: FUN-1, Pharmigen), HLA-DR (clone: AB3, Dako, Hamburg, Germany). Briefly, samples of 2×5 × 10^5 ^cells were resuspended in 50 μl PBS supplemented with 10% human immunoglobulin (Beriglobilin^®^, Centeon, Marburg, Germany). 5 μl of the antibodies were added to the cell suspension and incubated for 30 minutes at 4°C (1:10 dilution). The cells were washed, resuspended in 100 μl PBS and analyzed in a FACScan flow cytometer (BD Biosciences, Heidelberg, Germany). Propidium iodide (at 10 ng/ml) was used to identify non-viable cells. A total of 10.000 viable cells per sample were evaluated for specific staining. Results were analyzed using the WinMDI-software (Version 2.8, Joseph Trotter Scripps University, USA).

### Confocal microscopy

8-well-chamber LabTekII slides (Nunc, Wiesbaden, Germany) were coated with 0.01% Poly-L-Lysin (Sigma-Aldrich, Taufkirchen, Germany) for 5 min at 37°C and subsequently washed with PBS. DCs were resuspended in culture medium and attached to precoated slides for 20 min at 37°C at a density of 4 × 10^5 ^cells in 100 μl. Cells were fixed with 4% paraformaldehyde for 10 min at RT, permeabilized with Triton X-100 for 5 min, washed with PBS and blocked with 5% BSA/PBS for 20 min. F-actin was stained using Alexa488-fluorochrom conjugated Phalloidin (Molecular Probes, Karlsruhe, Germany) for 20 min in 1% BSA/PBS. After washing with PBS the slides were dried and mounted with Fluoromount-G medium (Southern Biotech, Biozol, Eching, Germany). Imaging was performed using Zeiss LSM 410 Meta confocal microscope, equipped with 63× oil objective (NA 1.4) and laser line 488 for excitation. For each analysis 100–200 DCs were randomly counted.

### Cytokine bead array (CBA)

Production of multiple cytokines by cultured DCs was simultanously determined using a CBA kit (BD Biosciences, Heidelberg, Germany). To this end, 50 μl of culture supernatant was incubated with a set of beads that have been coated with cytokine specific antibodies, which serves as a capture surface for cytokines. Each supernatant was analyzed in undiluted form as well as after diluting 1:10 and 1:100 in "Assay diluent" included in the CBA kit. Beads specific for IL-8, IL-10, IL-12, monocyte chemoattractant protein-1 (MCP-1) and for regulated on activation normal T-cell expressed and secreted (RANTES) were detected in a FACSCanto II flow cytometer (BD Biosciences) and the total amount of cytokines was then determined based on fluorescence intensity using the FCAP Array 1.0 software (Soft Flow).

### Mixed lymphocyte reaction (MLR)

T-cells were isolated from PBMCs by incubating them for 1 hour at 4°C with neuraminidase (Boehringer Ingelheim, Ingelheim, Germany) treated sheep red blood cells. Rosettes consisting of T cells were collected following a density gradient (see DC preparation) and prepared with 0.8% NH_4_Cl for lysis. Subsequently, remaining T cells were washed in PBS and resuspended in RPMI. The purity of the T cell preparations was assessed by flow cytometric analysis of CD3, CD4 and CD8 and routinely found to be around 98%. In order to achieve strong enough proliferation to allow measurement by a non-radioactive method, T cells were prepared following the "oxidative mitogenesis" protocols as published by Hill et al. 1999 [25]. In brief, purified T cells were incubated in 0,5 mM sodium periodide for 15 min on ice, washed in FCS followed by two washings in RPMI1640/10%FCS. To test the capacity of DCs to stimulate allogenic T cells, the DCs were cocultured in graded doses of 9000 to 111 cells per well with 9 × 10^4 ^allogenic sodium-periodide-treated T-cells for 3 days in U-bottom 96-well plates in a volume of 350 μl in RPMI1640/10%FCS/gentamycin. To determine the proliferation-index, the cultures were incubated with WST-8 cell proliferation array kit (Biozol, Eching, Germany) in a modified MTT assay for 2 hours. Optical density was analyzed at 450 nm using a microplate reader. 6–8 independent assays were performed for each condition.

### Data analysis

In total, 8–14 independent experiments were analyzed for each test system. Since the PBMC donors did not conform to a normal distribution pattern as assessed by the Shapiro-Wilkins test for normality, we used the Wicoxon Signed Rank test for statistical analysis of relative changes in cytokine concentration, and the nonparametric Mann Whitney U test for the comparison of surface marker expression, podosome formation and lymphocyte stimulatory capacity under the influence of the hormones. Statistical analyses refer to comparisons between hormonal treatments and the corresponding mDC sample. *P*-values < 0.05 were considered as significant (Graph-Pad Software, San Diego, USA).

## Results

Since the samples had been anonymized, the gender of the blood sample donors from which the DCs were derived was initially determined by PCR (AMXP gene analysis) according to Mitchell et al. [26]. Since no differences were observed between male (n = 6) and female (n = 8) donors the samples were combined. However, broad individual variations were observed in all parameters analyzed during the course of this study that were not related to the gender of the blood sample donors.

### Dex but not ActA and InA inhibit the expression of typical surface markers of mature DC

**Figure 1:**
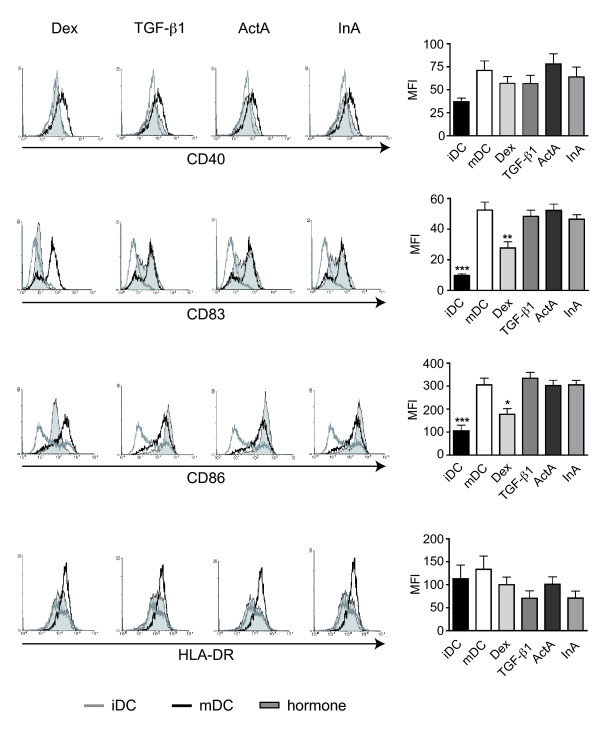
**Impact of ActA and InA on the maturation of DCs**. Immature dendritic cells (iDCs) were generated from PBMCs of healthy donors by culturing with IL-4 and GM-CSF. Differentiation into mature DCs (mDCs) was achieved by addition of an inflammatory cytokine cocktail for 24 hours. To study the impact of various hormones on the maturation process, maturation was performed in the presence or absence of Dex, TGF-β1, ActA or InA. Subsequently, the cells were analyzed for surface expression of CD40, CD83, CD86 and HLA-DR by flow cytometry. Representative histograms (left panel) are depicted for each hormonal treatment (grey shaded areas); analysis of corresponding iDCs (grey line) and mDCs (black line) are shown in each histogram for control. In addition, statistical analysis of the mean fluorescence intensity (MFI) as a quantitative measure for surface expression levels is depicted as bar diagrams (right panel). n = 14.

Firstly, we determined whether pretreatment with pregnancy-associated glycoprotein- hormones affected the phenotype of DCs during maturation. To this end, iDCs were stimulated with an inflammatory cocktail in the presence of the selected hormones and analyzed by flow cytometry for surface expression of molecules that characterize the differentiation stage of DCs. As known previously, iDCs express low levels of CD40, CD83, CD86 as well as HLA-DR. While CD40 and HLA-DR were only slightly upregulated during the maturation process, CD83 and CD86 became considerably induced on mature DCs, which was significantly prevented by Dex (Figure 1). In contrast, there was no clear effect on cell surface expression observed for any of the three glycoprotein hormones (Figure 1).

### Treatment with ActA and InA impacts on the F-actin cytoskeleton of DCs

**Figure 2:**
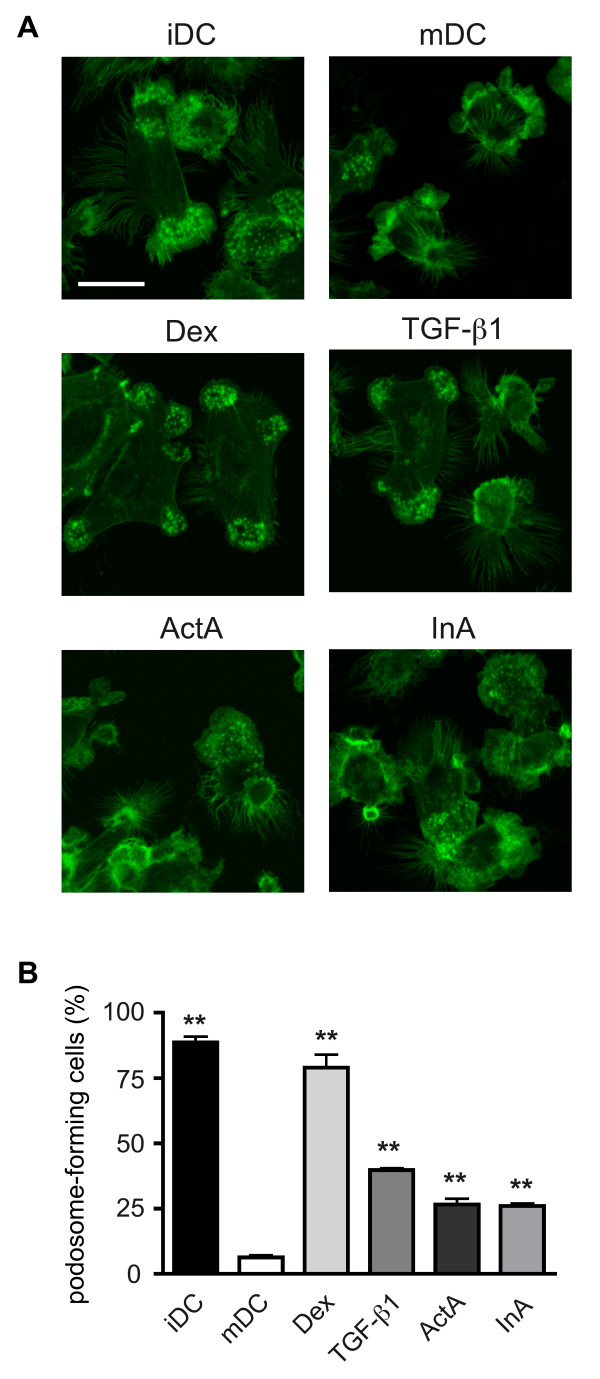
**Influence of ActA and InA on the F-actin cytoskeleton of DCs**. iDCs generated from PBMCs of healthy donors were differentiated into mDCs in the presence or absence of Dex, TGF-β1, ActA and InA. Changes in cell morphology were assessed by confocal microscopy following staining with Phalloidin-Alexa488 to visualize F-Actin structures. (A) Representative pictures of iDCs, mDCs and DCs matured in the presence of one of the four hormones are depicted; bar: 20 μm. (B) Quantification of the number of podosome-forming cells for each experimental condition as a measure of the degree of DC maturation. For each analysis 100–200 DCs were randomly counted. Mean +/- SEM; n = 5; ** p < 0.01, * p < 0.05 (hormonal treatment vs. mDC).

Since maturation of DCs is accompanied by dramatic changes in the cytoskeletal organization and morphology [27,28], we investigated the influence of hormonal treatment on F-actin remodeling by Phalloidin staining and confocal microscopy (Figure 2). The phenotype of iDCs was characterized by strongly enlarged cell bodies, podosomes and cell-substratum contact zones on the cell edges. In contrast, mDCs were round-shaped and displayed a complete loss of podosomal stuctures. Addition of Dex to iDC cultures during maturation fully prevented the adoption of a mature phenotype. This can be concluded from the morphological inspection of the cells and was confirmed by quantification of podosome-carrying cells (Figure 2A, B). Treatment with TGF-β1, ActA or InA also interfered with the maturation process by preveting F-Actin remodeling and inhibiting the dissolution of podosomes. However, the effects of the glycoprotein-hormones were less pronounced as compared to those of Dex (Figure 2).

### Cytokine and chemokine secretion are unaffected by ActA and InA

**Figure 3:**
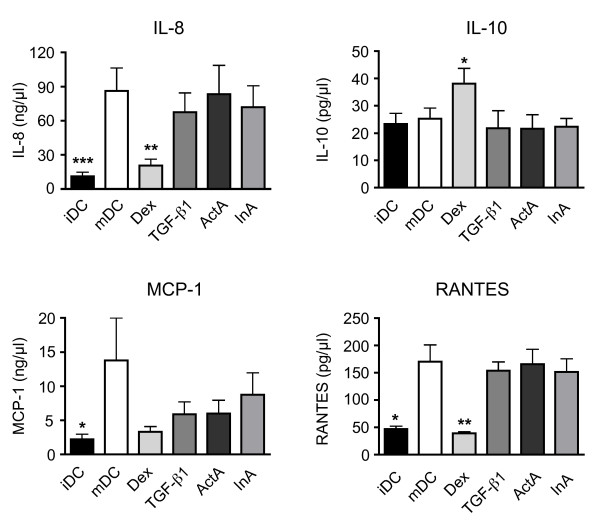
**Cytokine and chemokine secretion in the presence of ActA and InA**. The culture supernatant of iDCs induced to differentiate into mDCs in the presence or absence of the four hormones was analyzed by cytokine bead array for the production of IL-8, IL-10, MCP-1 and RANTES. Mean +/- SEM, n = 7- 8; ** p < 0,01 (hormonal treatment vs. mDC).

A major hallmark of mDCs is their spectrum of secreted mediators such as cytokine and chemokines that differs from the one of iDCs. Maturation of DCs by incubation with the inflammatory cocktail strongly increased the secretion of IL-8, MCP-1 and RANTES (Figure 3). In contrast, IL-10 levels did not change. Maturation in the presence of Dex significantly prevented up-regulation of IL-8 and RANTES secretion. Furthermore, IL-10 concentrations in the cell culture supernatant significantly increased in Dex treated cells. A strong impact of Dex on MCP-1 secretion was observed in all samples, however did not reach significance due to the large standard deviation. Neither incubation with TGFβ 1, ActA nor InA had any significant impact on the production of any of the mediators, even though we observed a non-significant down-regulation of MCP-1 secretion by TGF-β1, ActA and InA (Figure 3). Thus, none of the glycoprotein-hormones affects the release of the cytokines and chemokines tested herein following DC maturation.

### ActA and InA interfere with the T-cell stimulatory capacity of DCs

**Figure 4:**
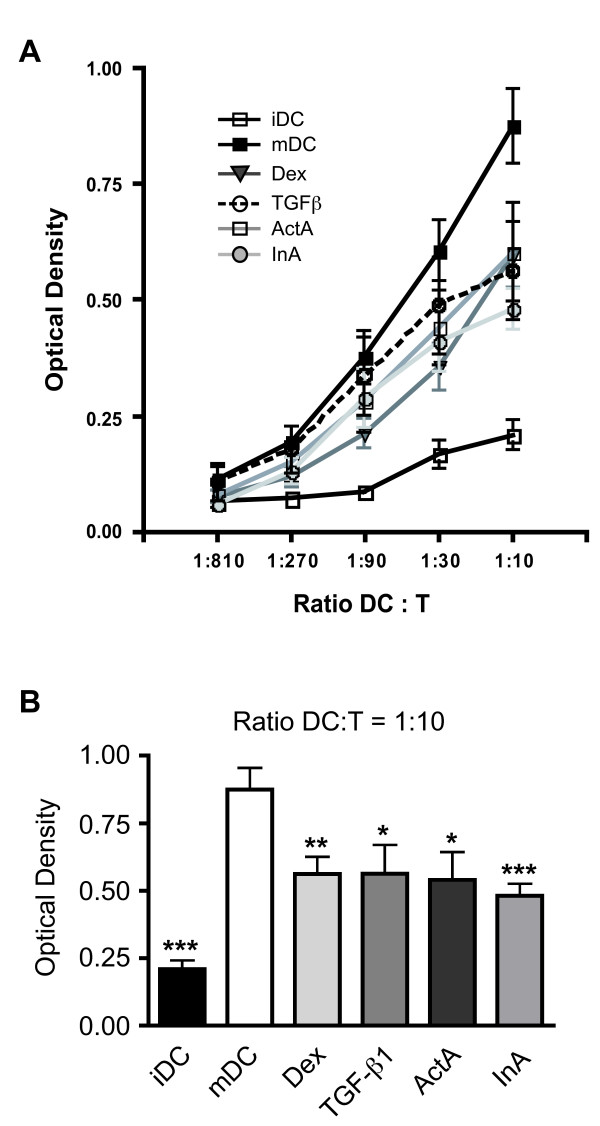
**T-cell stimulatory activity of DCs matured in the presence of ActA and InA**. **(A) **DC function was analyzed in a mixed lymphocyte reaction (MLR) by incubating DCs obtained under different culture conditions with T lymphocytes at the indicated ratios. T-cell proliferation induced by iDCs, mDCs or DCs matured in the presence of Dex, TGF-β1, ActA or InA was measured on the basis of the measured optical density as described in the methods section. Mean +/- SEM; n = 8. **(B) **At a DC:T ratio of 1:10, the reduction of the T-cell stimulatory capacity of the DCs was significantly reduced by all four hormones. Each bar represents n = 8 independent experiments.

Mixed lymphocyte reaction (MLR) experiments allow assessing the ability of DCs to serve as potent antigen presenting cells (APCs) during T-cell priming. As expected, the T-cell stimulatory capacity of iDCs was very low in comparison to that observed for mDCs. DCs matured in the presence of Dex were clearly impaired in their ability to induce T cell proliferation (Figure 4a). Similarly, TGF-β1, ActA and InA pre-treatment also resulted in a diminished T-cell stimulatory capacity of DCs, an effect that was comparable to the one of Dex (Figure 4a). At a DC:T ratio of 1:10, the capacity of matured DCs to induce allogeneic T cell stimulation was significantly reduced by all four hormones (Figure 4b). Thus, the pregnancy-associated glycoprotein-hormones not only interfere with the phenotype and morphology of DCs but also impair the function of DCs and their ability to serve as APCs during T-cell priming.

## Discussion

ActA and InA are known to play a key role in implantation and early embryo development [2-9]. Furthermore, ActA is also involved in inflammatory responses [29] and works as a chemoattractant for monocytes [30,31]. Recent studies revealed that ActA is able to modulate immune responses via type I and II activin receptors on DCs [32]. In early human pregnancy, a considerable recruitment of iDCs can be detected in human decidua compared to non-pregnant endometrium [14]. So far, it remains elusive whether ActA and InA exert immunomodulatory effects on the phenotype and function of DCs. Therefore we examined whether those two glycoprotein-hormones influence the maturation of DCs.

Since the inhibitory effect of Dex on the phenotype and function of DCs is well established [16-20], this was used as an internal control in all experiments. The doses of TGF-β1, ActA and InA used in the cell culture experiments were selected based previous publications [5,18,23]. In accordance with earlier studies [16,17] Dex treated DCs retained an apparently immature phenotype (CD40^low^, CD83^low^, CD86^low^, HLA-DR^low^). However, there was no clear effect of the three glycoprotein hormones on any of the four surface markers. This observation is in contrast to previous findings, which described a down-regulation of CD 40 and the costimulatory molecule CD86 by TGF-β1 in murine DC [23]. Proper functioning as antigen-presenting cells (APCs) requires DCs to change their morphology during the maturation process [28]. While iDCs are large cells with extensive lamellopodia and numerous podosomes, mDCs are much smaller and discontinue to exhibit those structures. Findings by others [33,34] and us demonstrate that glucocorticoids almost fully prevent the morphological alterations occurring during maturation and thereby interfere with the ability of DCs to serve as potent APCs. Here we showed that in the presence of the glycoprotein-hormones TGF-β1, ActA and InA, DCs also retained a partially immature phenotype although the effects were less strong in comparison to Dex. This suggests that TGF-β1, ActA and InA are able to modulate the maturation of DCs in terms of morphology.

The decidual micromilieu comprises a mixture of pro- and anti-inflammatory factors. It is believed that during pregnancy, pro-inflammatory factors support placentation by a self-destruction process of the decidual stroma [35,36] whilst anti-inflammatory cytokines rather induce a tolerant phenotype of DCs. ActA plays a complex role in various inflammatory processes. Since ActA is rapidly released into the circulation after intravenous injection of LPS [29], it likely represents a critical component of the early inflammatory pathway [37]. In line with this notion, ActA acts on key modulators of the inflammatory process such as TNF-α, IL-1β and IL-6 [29]. Furthermore, IL-6 and IL-8 production in amnion explants were found to be significantly stimulated by ActA [38] while it was inhibited at elevated levels of ActA [38]. In our system, neither TGF-β1, ActA nor InA significantly altered the production of pro- and anti-inflammatory chemokines and cytokines. As suggested by other groups, we therefore assume that ActA and InA rather work as genuine cytokines [37] and have only little impact on the direct modulation of mediator production by DCs. The suitability of our assay was confirmed by the finding that Dex strongly inhibited the production of the pro-inflammatory cytokine IL-8 and RANTES [29] as well as increased IL-10 synthesis [39].

Dex as the protagonist of an immunomodulatory hormone strongly interfered with surface expression, morphology and mediator production by DCs. In contrast, the three glycoprotein-hormones TGF-β1, ActA and InA exerted some but not all effects of Dex and also not to the same magnitude. Nevertheless, functional analysis of the T-cell stimulatory capacity of DCs matured in the presence of the four pregnancy-associated hormones clearly showed that all of them interfered with this important characteristic. TGF-β1, ActA and InA clearly reduced T-cell proliferation compared to mDCs. These findings are consistent with a recent report demonstrating that ActA attenuates the T-cell stimulatory capacity as measured by the expansion of antigen-specific CD8^+ ^T cells [32].

A general observation of all our experiments was that the effects of hormonal pre-treatment on DC maturation differed between individual donors. While some DC preparations showed very strong effects, others responded quantitatively weaker despite using standardized procedures. Since an impact of the gender of the donors could be excluded, we hypothesize that individual difference in the capacity of DCs to respond to maturation stimuli may exist. The effects of pre-incubation with TGF-β1, ActA, InA or Dex were most conspicuous when DCs were matured with the hormones for 24 hours. In contrast, addition to the cell cultures during differentiation of the monocytes into iDCs resulted in a deformation or even apoptosis of a large proportion of the cells (data not shown).

It is a well-known fact that the levels of ActA and InA in early pregnancy correlate with the success or failure of pregnancy but the underlying processes remain elusive. Our results now provide for the first time a rationale that these hormones are able to exert immunomodulatory functions that are important for the acceptance of the fetal semiallograft by supporting the development of tolerance-inducing DCs.

## Competing interests

The authors declare that they have no competing interests.

## Authors' contributions

SES drafted the manuscript, set up the experiments, participated in data collection, analysed and interpreted the results. NM carried out the confocal microscopy, analysed the results and provided images and figures. JB carried out the cytokine bead arrays, MK cultivated the cells and carried out the FACS analysis, JD participated in editorial support, HMR participated in design of the study, provided images and figures and revised the article for intellectual content. LR participated in the study design, experimental concept, and data interpretation UK participated in the study design, analysis and interpretation of the results and drafting of the manuscript. All authors read and approved the final manuscript.
